# Contribution of Maize Polyamine and Amino Acid Metabolism Toward Resistance Against *Aspergillus flavus* Infection and Aflatoxin Production

**DOI:** 10.3389/fpls.2019.00692

**Published:** 2019-05-24

**Authors:** Rajtilak Majumdar, Rakesh Minocha, Matthew D. Lebar, Kanniah Rajasekaran, Stephanie Long, Carol Carter-Wientjes, Subhash Minocha, Jeffrey W. Cary

**Affiliations:** ^1^Food and Feed Safety Research Unit, Southern Regional Research Center, United States Department of Agriculture, Agricultural Research Service, New Orleans, LA, United States; ^2^United States Department of Agriculture Forest Service, Northern Research Station, Durham, NH, United States; ^3^Department of Biological Sciences, University of New Hampshire, Durham, NH, United States

**Keywords:** *Aspergillus flavus*, *s*-adenosylmethionine decarboxylase, polyamine oxidase, mycotoxin, polyamine uptake, amino acids

## Abstract

Polyamines (PAs) are ubiquitous polycations found in plants and other organisms that are essential for growth, development, and resistance against abiotic and biotic stresses. The role of PAs in plant disease resistance depends on the relative abundance of higher PAs [spermidine (Spd), spermine (Spm)] vs. the diamine putrescine (Put) and PA catabolism. With respect to the pathogen, PAs are required to achieve successful pathogenesis of the host. Maize is an important food and feed crop, which is highly susceptible to *Aspergillus flavus* infection. Upon infection, the fungus produces carcinogenic aflatoxins and numerous other toxic secondary metabolites that adversely affect human health and crop value worldwide. To evaluate the role of PAs in aflatoxin resistance in maize, *in vitro* kernel infection assays were performed using maize lines that are susceptible (SC212) or resistant (TZAR102, MI82) to aflatoxin production. Results indicated significant induction of both PA biosynthetic and catabolic genes upon *A. flavus* infection. As compared to the susceptible line, the resistant maize lines showed higher basal expression of PA metabolism genes in mock-inoculated kernels that increased upon fungal infection. In general, increased biosynthesis and conversion of Put to Spd and Spm along with their increased catabolism was evident in the resistant lines vs. the susceptible line SC212. There were higher concentrations of amino acids such as glutamate (Glu), glutamine (Gln) and γ-aminobutyric acid (GABA) in SC212. The resistant lines were significantly lower in fungal load and aflatoxin production as compared to the susceptible line. The data presented here demonstrate an important role of PA metabolism in the resistance of maize to *A. flavus* colonization and aflatoxin contamination. These results provide future direction for the manipulation of PA metabolism in susceptible maize genotypes to improve aflatoxin resistance and overall stress tolerance.

## Introduction

Mycotoxin contamination of food and feed crops is a global threat. The major fungal genera that are the primary contributors of mycotoxin contamination in crop plants are *Aspergillus, Fusarium, Penicillium*, and *Alternaria*. Among these fungi, *Aspergillus flavus* has the most adverse impact on crop loss and human/animal health (Ismaiel and Papenbrock, 2015; Mitchell et al., 2016; Umesha et al., 2016). The fungus infects oilseed crops such as maize and peanut where it produces carcinogenic aflatoxins and other toxic secondary metabolites (SMs). Maize is a major food and feed crop grown worldwide. In 2013, economic losses resulting from *A. flavus* contamination in maize grown in the U.S. were estimated to be $686.6 million (Mitchell et al., 2016). The health impact of aflatoxin contamination is severe. Aflatoxin causes liver cancer, stunted growth, and *A. flavus* causes aspergillosis in immune compromised individuals (reviewed in Ojiambo et al., 2018). Abiotic stressors such as drought increase aflatoxin production in maize (Kebede et al., 2012; Fountain et al., 2014). The role of polyamines (PAs) in drought tolerance and tolerance to other abiotic stresses is well-established (reviewed in Minocha et al., 2014; Tiburcio and Alcázar, 2018). Therefore, drought tolerance and aflatoxin resistance could be useful traits in maize. A number of approaches to enhance aflatoxin resistance in maize are being examined that include conventional and marker-assisted breeding, transgenic expression of resistance-associated proteins, RNA-interference-based host induced gene silencing, and biocontrol (reviewed in Ojiambo et al., 2018).

**FIGURE 1:**
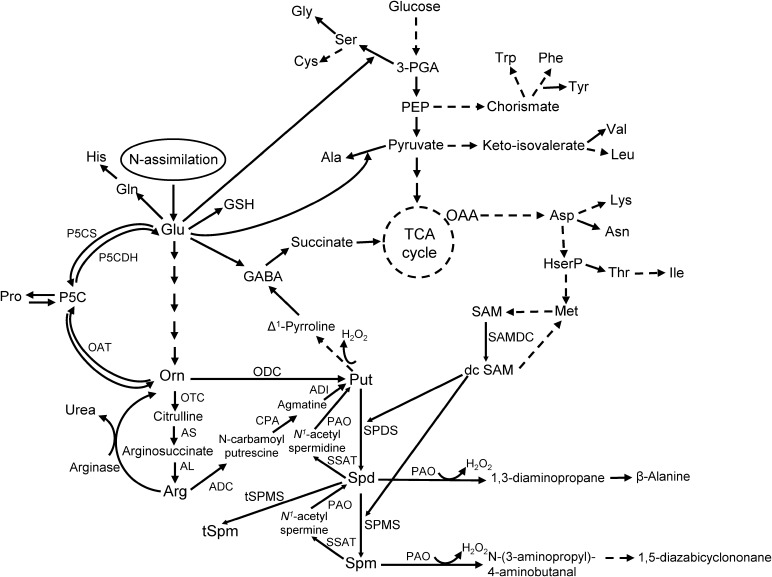
The pathway of polyamine metabolism and its connection with AA biosynthesis in plants (modified from Majumdar et al., 2013).

Polyamines are ubiquitous aliphatic amines found throughout all life forms. Their relative amounts vary depending on tissue type, developmental stage, or exposure to abiotic and biotic stressors (reviewed in Jiménez-Bremont et al., 2014; Minocha et al., 2014; Majumdar et al., 2017; Masson et al., 2017). The PA pathway (Figure 1) starts with the production of the diamine putrescine (Put) by the enzymes ornithine decarboxylase (ODC; E.C. 4.1.1.17) or arginine decarboxylase (ADC; E.C. 4.1.1.19) from the substrates ornithine (Orn) and arginine (Arg) respectively. Putrescine is converted into higher PAs, spermidine (Spd; a tri-amine) and Spm (Spm; a tetra-amine), by the enzymes Spd synthase (SPDS; E.C. 2.5.1.16) and Spm synthase (SPMS; E.C. 2.5.1.22) both of which require decarboxylated *S*-adenosylmethionine (dcSAM) produced by *S*-adenosylmethionine decarboxylase (SAMDC; E.C. 4.1.1.50). In animals, Spm and Spd are back-converted to Spd and Put, respectively, by Spm/Spd N^1^-acetyltransferase (SSAT; E.C. 2.3.1.57) and PA oxidase (PAO; E.C. 1.5.3.11); in plants, the SSAT is not well-characterized.

The PA pathway plays a critical role during normal development, stress response, and is biochemically linked to AA metabolism in plants (Majumdar et al., 2013, 2016; Minocha et al., 2014); especially the AAs Glu, Arg, Pro, Orn, and GABA. As Orn is synthesized from Glu, which serves both as an entry point for N, and a major precursor for several other AAs in plants, alterations in PA metabolism can impact AA levels in living cells (Mohapatra et al., 2010; Majumdar et al., 2013, 2016). The role of PAs in plant disease resistance is highly evident from several studies that involved alteration of tissue PA concentrations through genetic manipulations or exogenous application of PAs (reviewed in Jiménez-Bremont et al., 2014; Takahashi, 2016; Pal and Janda, 2017). Over-expression of a human *SAMDC* gene in tobacco resulted in increased accumulation of free and conjugated PAs and conferred tolerance to *Verticillium dahliae* and *Fusarium oxysporum* (Waie and Rajam, 2003). Transgenic eggplants over-expressing an oat *ADC* gene showed increased resistance against *Fusarium oxysporum*, the causal organism of wilt disease (Prabhavathi and Rajam, 2007). Transgenic plants accumulated both free and conjugated Put and Spd, and exhibited higher DAO activity than the control plants. Over-expression of other PA biosynthetic genes, *SPDS* and *SPMS* in sweet orange (*Citrus sinensis*) and *Arabidopsis thaliana*, respectively, increased resistance against bacterial pathogens (Fu et al., 2011; Gonzalez et al., 2011; Fu and Liu, 2013). Polyamine-associated disease resistance in plants is affected by an increase in free and conjugated PAs, catabolism of PAs, production of H_2_O_2_ and activation of defense signaling pathways. Other PA functions include up-regulation of genes involved in the production of pathogenesis-related (PR) proteins (especially by Spm; reviewed in Seifi and Shelp, 2019), transcription factors (e.g., basic leucine zipper protein family), and plant defense hormones, e.g., methyl-jasmonate (reviewed in Hussain et al., 2011; Jiménez-Bremont et al., 2014). Polyamines have also been implicated to indirectly lower the plant immune response against pathogenic bacteria (*Erwinia amylovora –* Oh et al., 2005, and *Pseudomonas syringae –* O’Neill et al., 2018) by the production of novel compounds like Phevamine A (a conjugate of L-Phe, L-Val, and a amidino-Spd).

Polyamines produced by the invading fungus are of importance with respect to growth, development, pathogenesis, and production of SMs (reviewed in Valdes-Santiago et al., 2012; Valdes-Santiago and Ruiz-Herrera, 2013). An *A. flavus Δspds* mutant failed to grow *in vitro* in the absence of exogenously supplied Spd in the growth medium and showed reduced growth and aflatoxin production during infection of maize kernels (Majumdar et al., 2018). In the WT *A. flavus* strain grown *in vitro*, an exogenous supply of Spd and Spm in the growth medium significantly increased fungal growth, sporulation, and production of aflatoxin and other toxic SMs, namely aflatrem, aflavinine, and cyclopiazonic (CPA) acid. Up-regulation of PAs in the host plant in response to a pathogen can be favorable to both host and pathogen. The outcome depends in large part on the relative abundance of the diamine Put versus higher PAs, Spd and Spm, accompanied by their catabolism, and also on the type of the pathogen and host species.

Knowing the diverse roles of PAs in plant disease resistance, the current study was undertaken to investigate the role of host PA metabolism during maize-*A. flavus* pathogenic interaction. The work described here, used maize lines that were previously characterized as either resistant or susceptible to *A. flavus* infection and aflatoxin contamination. The genotypes used in this current study were selected based on the aflatoxin data obtained from multiyear field studies (reviewed in Brown et al., 2016). The data presented here demonstrate a significant induction of Put biosynthetic genes accompanied by greater conversion of Put into higher PAs (Spd and Spm) in resistant lines in comparison with a susceptible maize line. In mock-inoculated kernels, higher basal expression of PA metabolism genes in resistant, as compared to susceptible lines, may form the basis for future breeding or transgenic approaches to improve aflatoxin resistance and overall stress tolerance in maize. In addition, higher Spd and/or Spm content could possibly be used to screen maize genotypes for potential aflatoxin resistance.

## Materials and Methods

### Maize Kernel Inoculation and Incubation

Undamaged and uniformly sized kernels of one susceptible genotype, SC212, and two resistant genotypes (TZAR102 – highly resistant, and MI82 – moderately resistant; Brown et al., 2016) of maize were processed for the kernel screening assay (KSA) as described in Rajasekaran et al. (2013). Briefly, kernels were surface sterilized with 70% ethanol, air dried, and kept sterile until experiments were started. The AF13 strain of *A. flavus* [SRRC 1532, a highly pathogenic and high aflatoxin-producing isolate (Cotty, 1989)] was grown on V8 agar medium for 7 days under illumination at 30°C prior to the collection of spores for kernel inoculation. Sterile kernels were inoculated by placing in a sterile 300 ml beaker containing 100 ml of AF13 spore suspension (4 × 10^6^ spores/ml) for 3 min with continuous stirring. Excess inoculum was removed and the kernels were placed in plastic caps arranged in trays containing filter paper on the bottom. The filter paper was moistened by addition of sterile ddH_2_0 and kept moist during the course of the experiment to maintain high relative humidity. *Aspergillus flavus* inoculated or water-inoculated (control, “mock-inoculated”) kernels were placed inside trays (with lids on top) and kept in an incubator at 31°C in the dark. Kernels were collected at 8 h, 3 and 7 days post-inoculation.

### Quantification of Polyamines and Amino Acids

Infected or control (water inoculated) ground maize kernels previously stored at -80°C were subjected to repeated (3X) freeze (-20°C) and thaw (room temperature) cycles in 5% PCA. The samples were then vortexed for 2 min after final thaw and centrifuged at 14,000 × *g* for 8 min. Dansylation and quantification of PAs and AAs was performed simultaneously using the method as described in Minocha and Long (2004) with minor modifications (Majumdar et al., 2018). The samples were incubated for 30 min at 60°C followed by cooling for 3 min and centrifuged for 30 s at 14,000 × *g*. The reaction was terminated by adding glacial acetic acid. Microfuge tubes containing samples were kept open under a flow hood for 3 min for evaporation of CO_2_. Acetone used to solubilize dansyl chloride was evaporated in a SpeedVac Evaporator (Savant, Farmingdale, NY, United States) for 5 min. Filtered HPLC grade methanol was added to each sample for a final volume of 2 ml.

The HPLC system was comprised of a Series 200 pump, auto-sampler, and fluorescence detector (Perkin-Elmer Corporation, Waltham, MA, United States) fitted with a 200 ml injection loop (20 ml injection volume). A column heater (Bio-Rad Laboratories, Hercules, CA, United States) was set at 40°C. The other components of the HPLC system included a Perkin-Elmer-Brownlee Pecosphere scavenger cartridge column (CRC18, 3 mm, 33 mm × 4.6 mm I.D.), a Phenomenex Securityguard^TM^ guard column (C18, 5 mm, 3 mm × 4 mm I.D.; Phenomenex, Torrance, CA, United States), and a Phenomenex Synergi^TM^ Hydro-RP analytical column (C18, 80 Å, 4 mm, 150 mm × 4.6 mm I.D.). Excitation and emission wavelengths were set at 340 and 515 nm respectively, and the data were processed using Perkin Elmer TotalChrom software (version 6.2.1).

### Aflatoxin and Polyamine Conjugates Analysis

Ground maize kernels (∼20–70 mg) inoculated with either AF13 strain or water (control) were extracted in 1 ml of methanol for 24 h with shaking (175 rpm) at room temperature. The extracts were filtered using cotton plugs and the filtrates dried under a stream of nitrogen. Extracts were reconstituted in 250 μl of methanol, centrifuged to remove particulates, and analyzed in a Waters Acquity UPLC system (Waters Corporation, Milford, MA, United States) (isocratic separation with 40% methanol in water, and a BEH C18 1.7 μm, 2.1 mm × 50 mm I.D. column) using fluorescence detection (excitation at 365 nm, and emission at 440 nm). Samples were diluted 10-fold if the aflatoxin signal saturated the detector. Analytical standards (Sigma-Aldrich, St. Louis, MO, United States) were used to identify and quantify aflatoxins [retention time of aflatoxin B1 (AFB1), 4.60 min; retention time of aflatoxin B2 (AFB2), 3.55 min]. Aflatoxin content was expressed as ng/mg fresh weight (FW) of homogenized kernels. Putrescine and Spd conjugates were analyzed on a Waters Acquity UPLC system equipped with a PDA UV detector and an Acquity QDa mass detector using the following conditions: solvent A = 0.1% formic acid (FA) in water; solvent B = 0.1% FA in acetonitrile; flow rate: 0.5 ml/min; solvent gradient: 5% B (0–1.25 min), gradient to 25% B (1.25–1.5 min), gradient to 100% B (1.5–5.0 min), 100% B (5.0–7.5 min), then re-equilibration to 5% B (7.6–10.1 min). Putative Put and Spd conjugates were identified by their molecular ion and corresponding UV spectrum [*N′,N″*-di-feruloyl-Put: 2.85 min (M+H)^+^ = 441.2 *m/z*, λ_max_ = 218.5, 235.0, 293.3, 317.9; *N′,N″*-di-coumaroyl Spd: 2.50 min (M+H)^+^ = 438.2 *m/z*, λ_max_ = 212.7, 225.8, 297.6, 307.4]. Quantification of *N′,N″*-di-feruloyl-Put and *N′,N″*-di-coumaroyl Spd was achieved by peak integration of the extracted ion chromatograms and normalized by sample weight.

### RNA Isolation, cDNA Synthesis, and Gene Expression

Total RNA from infected or control (water-inoculated) ground maize kernels was isolated using a ‘Spectrum^TM^ Plant Total RNA kit’ (Sigma-Aldrich). cDNA was synthesized using an iScript^TM^ cDNA synthesis kit (Bio-Rad). Manufacturer’s protocols were followed for both RNA isolation and cDNA synthesis. Quantitative RT-PCR (qRT-PCR) was performed in an iCycler iQ5 Multicolor real-time PCR detection system (Bio-Rad) using SYBR green I chemistry. The thermocycler conditions comprised of a pre-incubation step at 95°C for 3 min, dye activation at 95°C for 10 s, primer annealing at 55°C for 30 s, elongation at 55°C for 50 s, and a dissociation curve between 65 and 95°C for 30 min (with 0.5°C increments). The primers used for the qRT-PCR analyses are listed in Supplementary Table S1. Gene expression was normalized by ΔΔ*C*_T_ method (Livak and Schmittgen, 2001) to *Zea mays* ribosomal structural gene GRMZM2G024838 or *A. flavus* β-*tubulin* gene (AFLA_068620) expression (Shu et al., 2015) using the gene expression analysis software package of the Bio-Rad iQ5.

Fungal loads in the infected maize kernels were estimated at 8 h, 3 and 7 days post *A. flavus* infection. Quantification of fungal load was performed according to Thakare et al. (2017), and calculated as relative expression of *A. flavus* β*-tubulin* gene (AFLA_068620) to the expression of maize ribosomal structural gene GRMZM2G024838 (Shu et al., 2015).

### Statistical Analysis

Student’s *t*-test was performed to determine statistical significance between the *A. flavus* susceptible line (SC212) and the resistant maize genotypes (TZAR102 and MI82), and between mock-inoculated and *Af*-inoculated within each line at a specific time point. The level of significance was determined at *P* ≤ 0.05 and is depicted in the figure legends and graphics as ^∗^ and # respectively.

## Results

### Polyamine Content

**FIGURE 2:**
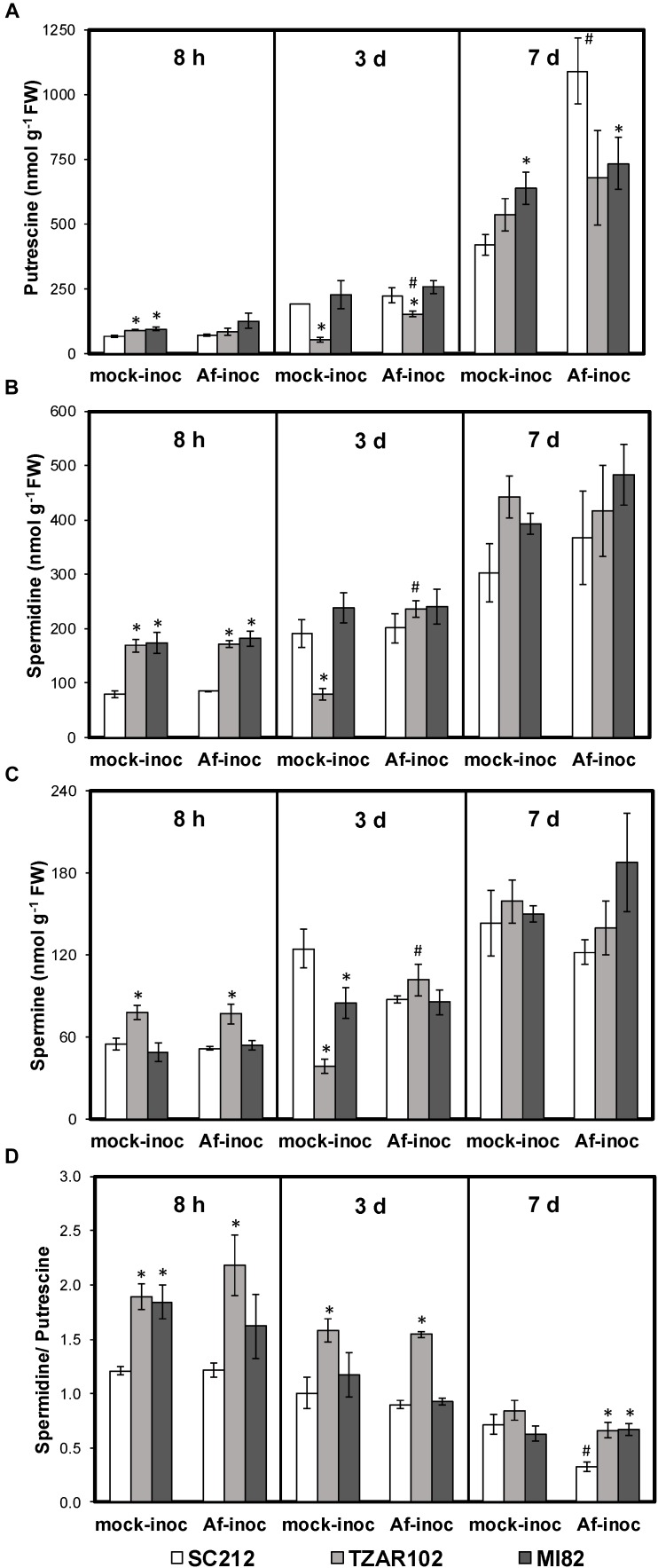
Altered polyamine metabolism in the susceptible (SC212) and resistant (TZAR102, MI82) maize genotypes. Cellular content of **(A)** putrescine; **(B)** spermidine; **(C)** spermine; **(D)** spermidine/putrescine ratio at different times post-inoculation (pi) in the mock-inoculated (mock-inoc) and *Aspergillus flavus* inoculated (*Af*-inoc) kernels of susceptible (SC212) and resistant (TZAR102, MI82) maize genotypes. Data are Mean ± SE of 4 replicates, each replicate consists of six seeds (^∗^*P* ≤ 0.05, between the susceptible line SC212 and other lines; ^#^*P* ≤ 0.05, between mock and +*Af* treatments within each line at different times after inoculation).

Polyamine content varied with incubation period, and with or without *A. flavus* infection between the resistant and susceptible maize lines. At 8 h mock-inoculated samples of resistant TZAR102 and MI82 lines had 35–43% higher Put content than the susceptible line (Figure 2A). No significant change in Put content was observed among *Af*-inoculated kernels of resistant and susceptible lines at this time point. At 3 days post-inoculation (dpi), Put content was significantly lower in the inoculated TZAR102 line (152 ± 10 nmol/g FW) as compared to the susceptible line SC212 (223 ± 30 nmol/g FW). Put content was significantly higher in the *Af*-inoculated TZAR102 line in comparison to the mock-inoculated kernels at this time point. At 7 dpi, Put content increased in all lines in both *Af*-inoculated and mock-inoculated kernels. At 7 dpi, Put content was highest in the *Af*-inoculated kernels of SC212 (1092 ± 129 nmol/g FW); 33–49% higher than the resistant lines, and significantly higher than the SC212 mock-inoculants. A 160% increase in Put content was observed in the *Af*-inoculated kernels of SC212 line in comparison to the mock-inoculated kernels at this time point.

Cellular content of Spd was significantly higher (102–117%) at 8 h (Figure 2B) in both mock and *Af*-inoculated TZAR102 and MI82 lines in comparison with SC212. There were no major differences in Spd between the lines at 3 or 7 dpi.

With respect to Spm, its content was significantly higher (∼45%) in TZAR102 (vs. SC212) at 8 h, in both the *Af*- and mock-inoculated samples (Figure 2C). No major change in Spm content was observed at 3 and 7 dpi in the susceptible and resistant lines, except for relatively lower levels in mock-inoculated TZAR102 and MI82 samples in comparison with SC212 at 3 dpi. In the TZAR102 line at 3 dpi, there was a significant increase in all three of the polyamines in the *Af*- vs. mock-inoculated samples.

In comparison with the SC212 susceptible line, the ratio of Spd/Put was significantly higher (33–79%) in the mock-inoculated kernels of MI82 and in the mock- and *Af*-inoculated kernels of TZAR102 (both resistant lines) at 8 h (Figure 2D). The TZAR102 line maintained higher Spd/Put ratio in both groups of inoculants (vs. SC212) at 3 days. At 7 days the Spd/Put ratio was significantly higher (102–104%) in the *Af*-inoculated kernels of TZAR102 and MI82 lines than in those from the SC212 line, and the *Af*-inoculated kernels of SC212 had a Spd/Put ratio that was significantly less than mock-inoculated kernels. For all lines the ratio of Spd/Put decreased over time.

### Changes in Put and Spd Conjugates in Response to *A. flavus* Infection

**FIGURE 3:**
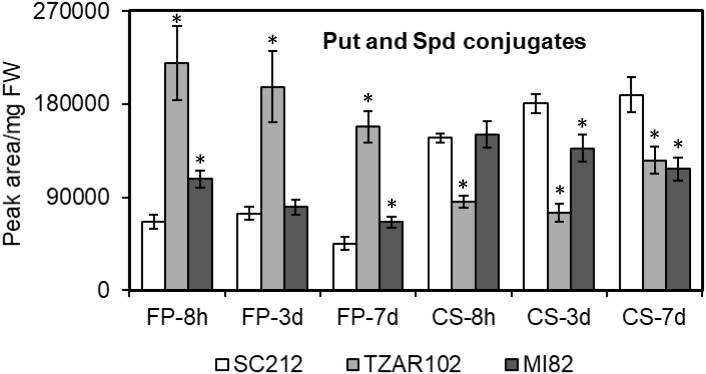
Putrescine and spermidine conjugates detected are differentially affected in the susceptible (SC212) and resistant (TZAR102, MI82) maize genotypes. Cellular contents of *N′,N″*-di-feruloyl-putrescine (FP) and *N′,N″*-di-coumaroyl spermidine (CS) in the *A. flavus* inoculated kernels at 8 h, 3 and 7 days post-infection. Data are Mean ± SE of 5 replicates, each replicate consists of six seeds (^∗^*P* ≤ 0.05, between the susceptible line SC212 and other lines at different times after inoculation).

Polyamine conjugates are known to have antimicrobial properties; we therefore investigated PA conjugates in the susceptible and resistant maize genotypes during *A. flavus* infection. The two maize PA conjugates that were detected in the current study were *N′,N″*-di-feruloyl-Put (FP) and *N′,N″*-di-coumaroyl Spd (CS) (Figure 3). The FP content was ∼60–230% higher in the resistant lines as compared to SC212 at 8 h. Between the two resistant lines, TZAR102 maintained a higher level (by 100–200%) of FP content throughout the infection period as compared to the other lines. The CS content on the other hand was similar at 8 hpi in both resistant and susceptible lines except for TZAR102, which was significantly lower than the other lines. At 3 and 7 dpi, CS content was decreased by 38–59% in the resistant lines in comparison to SC212.

### Amino Acids Analyses

**FIGURE 4:**
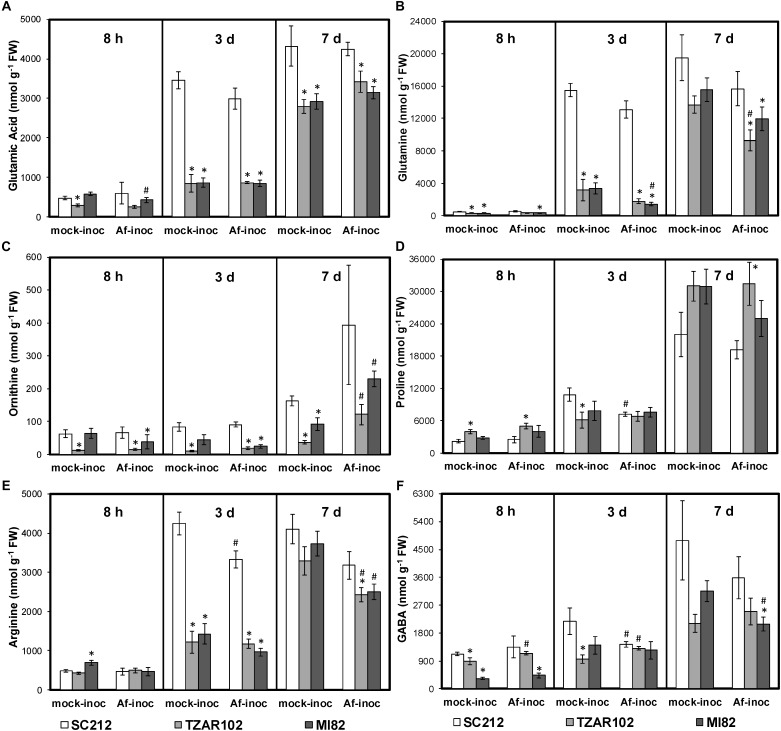
Amino acids are differentially regulated in the susceptible vs. resistant lines. Cellular contents of **(A)** glutamate, **(B)** glutamine, **(C)** ornithine, **(D)** proline, **(E)** arginine, and **(F)** γ-aminobutyric acid (GABA) at 8 h, 3 and 7 days in the mock-inoculated (mock-inoc) and *A. flavus* inoculated (*Af*-inoc) kernels of susceptible (SC212) and resistant (TZAR102, MI82) maize genotypes. Data are Mean ± SE of 4 replicates, each replicate consists of six seeds ^∗^*P* ≤ 0.05, between the susceptible line SC212 and other lines; ^#^*P* ≤ 0.05, between mock and +*Af* treatments within each line at different times after inoculation).

As cellular Put is produced from the substrates Orn and Arg, both of which are derived from Glu (a key precursor of AAs), any change in Orn and Arg utilization will affect related AAs (Figure 1). There were no major changes in cellular levels of Glu in the resistant and susceptible lines at 8 hpi except for TZAR102, which had significantly lower Glu in the mock-inoculated samples as compared to SC212 mock-inoculants (Figure 4A). At 3 dpi Glu content increased by 100% in both the mock and *Af*-inoculated SC212 line with a further increase in both at 7 dpi. At days 3 and 7 Glu content was significantly higher in both SC212 inoculants than all other inoculants of the susceptible and resistant lines.

The differences in cellular Gln content between the susceptible and the resistant lines were relatively small at 8 dpi, however Gln in both of the resistant lines was significantly lower in the mock-inoculated and the *Af*-inoculated MI82 samples than in the susceptible SC212 line. At 3 dpi, cellular Gln content was significantly higher in SC212 relative to the other two lines for both *Af*-inoculated and mock-inoculated samples (Figure 4B). The trend remained the same for inoculated samples at 7 days.

Cellular Orn content in the *Af*-inoculated MI82 and *Af*- and mock-inoculated TZAR102 lines was significantly lower than the susceptible line SC212 at 8 h and 3 dpi (Figure 4C). At 7 dpi, Orn in the mock inoculants of both resistant lines was significantly lower than the mock-inoculated susceptible line and the *Af*-inoculated samples of the same lines. Mock-inoculated TZAR102 samples had significantly lower Orn relative to SC212 at all times tested.

As Pro is derived from Orn, any change in cellular Orn content will affect Pro content. In TZAR102, cellular content of Pro was significantly higher (100–144%) in mock- and *Af-*inoculated samples at 8 hpi, and 7 dpi *Af*-inoculated samples when compared to SC212 (Figure 4D). At 3 dpi, Pro was significantly lower in the TZAR102 mock-inoculated kernels, and significantly higher at 7 dpi in *Af*-inoculated TZAR102 vs. SC212.

Cellular Arg content increased by 600% from 8 hpi to 3 dpi in both inoculants of SC212, and was significantly higher than all other susceptible and resistant lines at this time point (Figure 4E). At 7 dpi, in comparison with mock-inoculated samples, Arg content was significantly lower in the *Af*-inoculated samples of both resistant lines.

The non-protein AA GABA is produced in two ways, via catabolism of Put and directly from Glu by Glu decarboxylase (Figure 1). At 8 hpi, cellular contents of GABA were significantly lower in the mock-inoculated samples of TZAR102, and both inoculants of the MI82 kernels in comparison to SC212 (Figure 4F). At 3 dpi this trend was seen only for mock-inoculated TZAR102 line. Cellular GABA content increased by >200% at 7 dpi (vs. 8 hpi and 3 dpi time points) in SC212 and was significantly higher than the *Af*-inoculated MI82 line. For the inoculated samples, there were no significant differences in GABA between the susceptible line SC212 and resistant line TZAR102 at any time tested (Figure 4F).

The data on AAs that are not directly related to the Glu-Orn-Arg-Pro-Put pathway, are presented in the Supplementary Data (Supplementary Figure S1). In general, the AAs that were decreased by >63–92% both in mock-inoculated and *Af*-inoculated samples of TZAR102 and MI82 resistant lines were: His, Ser, Leu, Ala, Val, Ile, Gly, Met, and Thr. The majority of these decreases were seen at 3 dpi.

### Expression of Polyamine Genes in Maize

**FIGURE 5:**
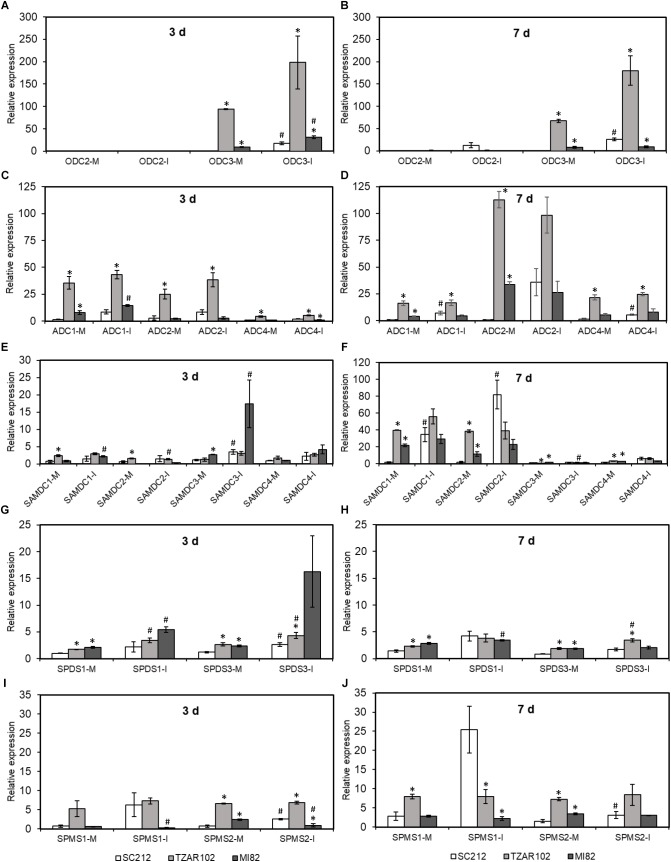
Polyamine biosynthetic genes are highly induced by *A. flavus*. Expression of maize polyamine biosynthetic genes, **(A,B)** *ODC*, **(C,D)** *ADC*, **(E,F)** *SAMDC*, **(G,H)** *SPDS*, **(I,J)** *SPMS* at 3 and 7 days in the mock-inoculated (mock-inoc) and *A. flavus* inoculated (Af-inoc) kernels of susceptible (SC212) and resistant (TZAR102, MI82) maize genotypes. Data are Mean ± SE of 3 replicates, each replicate consists of six seeds (^∗^*P* ≤ 0.05, between the susceptible line SC212 and other lines; ^#^*P* ≤ 0.05, between mock and +*Af* treatments within each line at different times after inoculation).

Biosynthesis of Put takes place via *ODC* and *ADC* pathways. Among the three *ZmODC* genes, expression of *ZmODC3* was highest in the TZAR102 resistant line. With one exception, mock and *Af*-inoculated kernels of both resistant lines had significantly higher expression of *ZmODC3* than the susceptible SC212 line at 3 and 7 dpi (Figure 5A,B). Expression of *ZmODC3* was highly up-regulated upon fungal inoculation at 3 and 7 dpi in SC212 and MI82 lines. Expression of *ZmODC2* was lower than *ZmODC3* in all lines at both time points with no differences between lines or inoculants. No expression of *ZmODC1* was observed in any of the samples.

The expression of four putative *ZmADC* genes varied between the susceptible and resistant lines at 3 and 7 dpi (Figure 5C,D). Expression of *ZmADC1* was significantly higher (5 to 24-fold) in both inoculants of the TZAR102 line compared to the SC212 line at 3 and 7 dpi. At 3 dpi in the MI82 line and 7 dpi in the SC212 line, *ZmADC1* expression was relatively higher in the *Af*-inoculants than the mock-inoculants. Expression of *ZmADC2* expression was 9 to 12-fold higher in the mock-inoculated TZAR102 kernels at 3 dpi, and 3 to 110-fold higher at 7 dpi in comparison to the SC212 line. At both 3 and 7 dpi mock inoculants of both resistant lines had significantly higher levels of *ZmADC2* expression than the susceptible line. Expression of *ZmADC4* was significantly higher in both mock and *Af*-inoculated kernels of TZAR102 as compared to SC212 at both 3 and 7 dpi. In comparison with mock-inoculants, *Af*-inoculants of SC212 had significantly higher levels of expression of *ZmADC4* at 7 dpi. No expression of *ZmADC3* was observed in any of the lines.

Among the four putative *ZmSAMDC* genes, *ZmSAMDC1* expression was 3 to 25-fold higher in the mock-inoculated kernels of TZAR102 line at 3 and 7 days, and MI82 at 7 days in comparison to the susceptible line (Figure 5E,F). Expression of *ZmSAMDC1* and *ZmSAMDC2* increased by ≥20-fold in the *Af*-inoculated samples at 7 dpi in comparison to their corresponding values at 3 dpi. At 3 days, *ZmSAMDC3* showed higher induction in the inoculated samples than their corresponding mock-inoculated controls in the susceptible SC212 and resistant MI82 lines. At 7 days, *Af*-inoculated SC212 kernels showed >50-fold upregulation of *ZmSAMDC1 and ZmSAMDC2* expression vs. their corresponding mock-inoculated samples.

Overall, relative induction of *ZmSPDS* and *ZmSPMS* genes (upon *Af*-inoculation) was substantially lower than all other PA biosynthetic genes (Figure 5G–J). Expression of *ZmSPDS1* and *ZmSPDS3* were significantly higher in the mock-inoculated kernels of TZAR102, and MI82 lines (vs. SC212) and was up-regulated upon fungal infection at 3 dpi (Figure 5G). At 7 dpi, the trend of *ZmSPDS3* expression in the resistant lines was similar to 3 dpi and was up-regulated in the TZAR102 line upon fungal inoculation (Figure 5H). No expression of the *ZmSPDS2* gene was observed in any of the samples. Expression of *ZmSPMS2* was up-regulated in the susceptible SC212 line both at 3 and 7 days upon fungal infection (Figure 5I,J). Expression of both *ZmSPMS1* and *ZmSPMS2* was highest in the mock-inoculated TZAR102 kernels both at 3 and 7 days and maintained similar level of expression during fungal infection.

**FIGURE 6:**
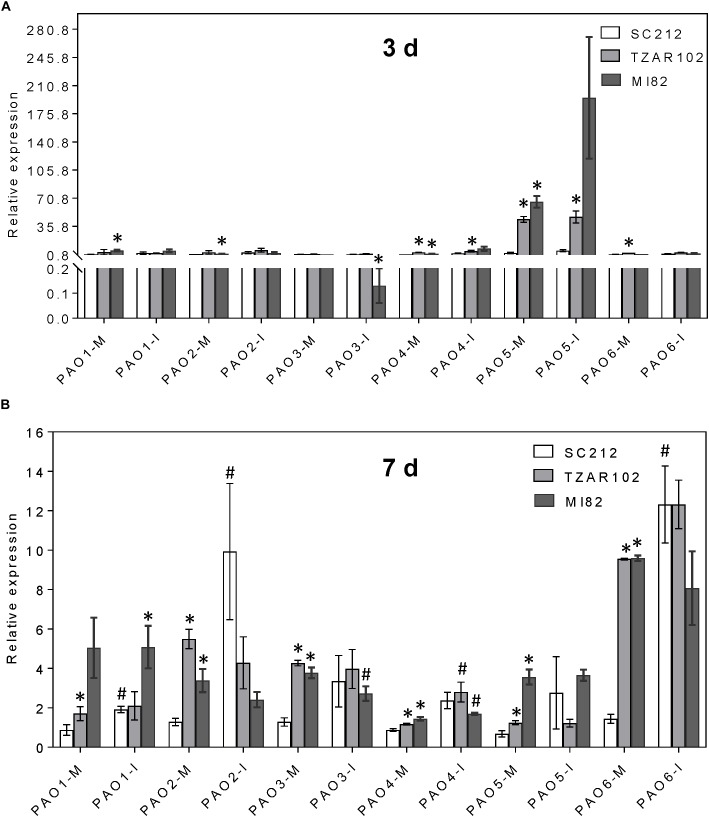
Polyamine catabolism genes are highly induced by *A. flavus*. Expression of maize polyamine catabolism gene, PAOs, at **(A)** 3 days, and **(B)** 7 days in the mock-inoculated (mock-inoc) and *A. flavus* inoculated (*Af*-inoc) kernels of susceptible (SC212) and resistant (TZAR102, MI82) maize genotypes. Data are Mean ± SE of 3 replicates, each replicate consists of six seeds (^∗^*P* ≤ 0.05, between the susceptible line SC212 and other lines; ^#^*P* ≤ 0.05, between mock and +*Af* treatments within each line at different times after inoculation).

Increased expression of Spd and Spm biosynthetic genes affected the expression of *ZmPAO* genes (Figure 6). Among the six *ZmPAO* genes, the pattern of relative change in *ZmPAO1-3* expression (mock-inoculated vs. *Af*-inoculated) was similar in all lines. Expression of *ZmPAO5* at 3 days was 50 to 100-fold higher in the TZAR102 and MI82 resistant lines vs. SC212 susceptible lines in both inoculants (Figure 6A). At 3 and 7 dpi, expression of *ZmPAO4-6* was higher in the mock-inoculated kernels of resistant lines in comparison with the susceptible line SC212 (Figure 6B). There was a sevenfold increase in *ZmPAO6* expression in *Af*-inoculated SC212 than the mock-inoculated samples.

### Fungal Load and Aflatoxin Production

**FIGURE 7:**
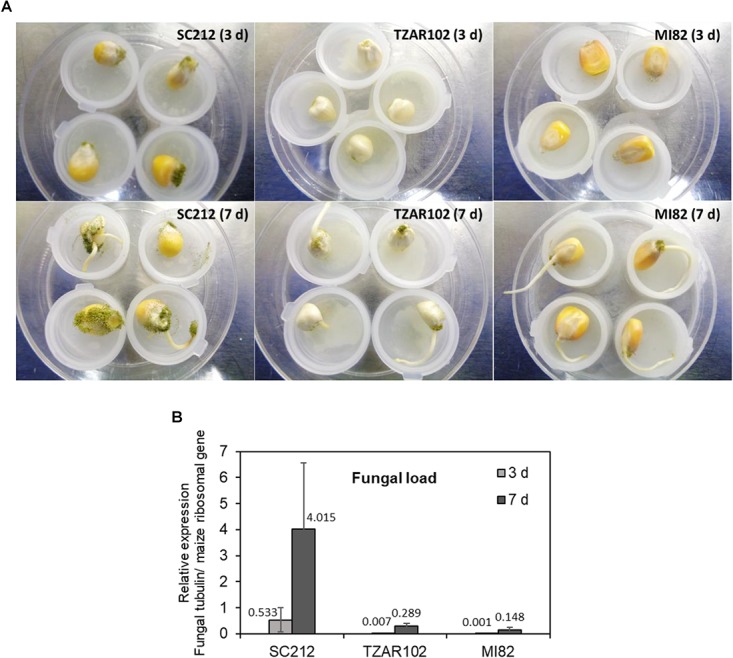
Fungal growth varies between susceptible vs. resistant maize genotypes. **(A)** Representative pictures of *A. flavus* colonization on the kernels of susceptible (SC212), and resistant (TZAR102 and MI82) maize genotypes at 3 and 7 days post-inoculation during an *in vitro* seed infection assay; and **(B)** quantification of fungal load at 3 and 7 days in the *A. flavus* inoculated kernels of susceptible and resistant maize genotypes. Expression of *A. flavus β-tubulin* gene (AFLA_068620) was normalized to the expression of maize ribosomal structural gene (GRMZM2G024838; Shu et al., 2015). Data are Mean ± SE of 3 replicates, each replicate consists of six seeds.

The susceptible line (SC212) showed higher *A. flavus* colonization on the kernels at 3 dpi and increased by 7 dpi as compared to the TZAR102 and MI82 resistant lines (Figure 7A). At both 3 and 7 dpi, the susceptible line had higher fungal load than the resistant lines (Figure 7B). Eight hpi data were inconsistent and are not presented.

**FIGURE 8:**
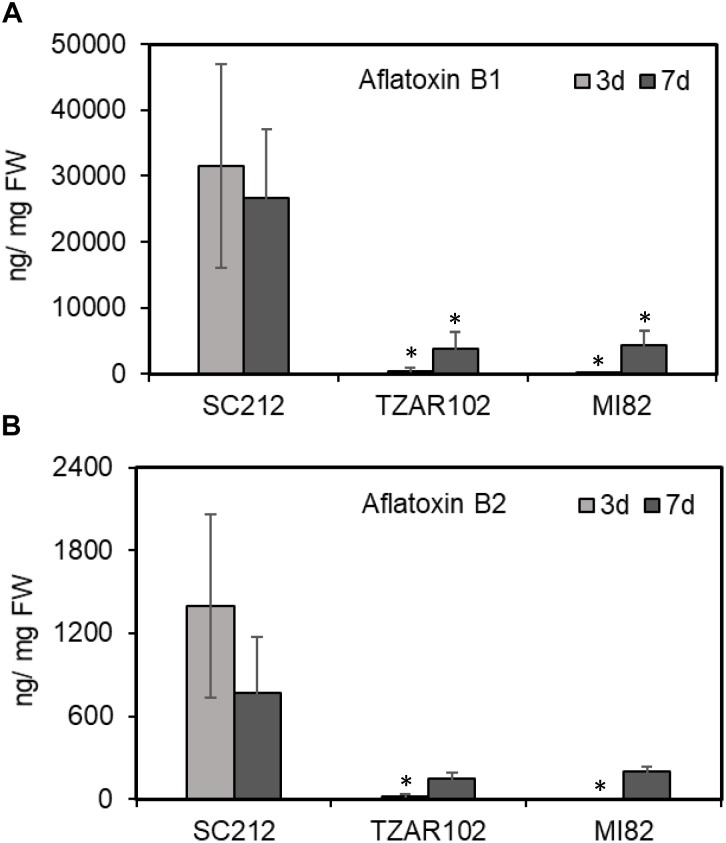
Aflatoxin content in the kernels of susceptible (SC212) and resistant (TZAR102, MI82) maize genotypes at 3 and 7 days post *A. flavus* infection. **(A)** Aflatoxin B1; and **(B)** aflatoxin B2. Data are Mean ± SE of 3–4 replicates, each replicate consists of 6 seeds (^∗^*P* ≤ 0.05; between the susceptible line SC212 and other lines, Student’s *t*-test).

In general, aflatoxin B1 (AFB1) content was several fold higher than aflatoxin B2 (AFB2; Figure 8) in all lines. No aflatoxin was detected in any samples at 8 hpi. The susceptible SC212 line accumulated the highest amount of aflatoxins at both 3 and 7 dpi. At 3 dpi AFB1 content in SC212 kernels was significantly higher than in both inoculants of the resistant lines TZAR102 and MI82. Aflatoxin content was significantly higher at 7 dpi in the resistant lines vs. 3 dpi, but it was still significantly lower than the susceptible line. The trend was similar with AFB2 content in the infected kernels of susceptible and resistant maize lines.

## Discussion

Polyamines are present in all life forms and are involved in a plethora of cellular processes including growth, development, stress response, and pathogenesis (reviewed in Valdes-Santiago et al., 2012; Minocha et al., 2014; Miller-Fleming et al., 2015; Takahashi, 2016; Pal and Janda, 2017). Consequently, the PA metabolic pathway in the host plant as well as in the pathogen has been the target of a number of studies to improve disease resistance in plants.

### Polyamine Biosynthesis, Conversion, and Catabolism Play an Important Role in Resistance to *A. flavus*

Polyamines are widely implicated in plant defense or susceptibility to disease (depending on the type of PAs and their relative abundance) during interaction with pathogens and pests (Subramanyam et al., 2015; Takahashi, 2016; Pal and Janda, 2017). Often, transgenic expression of the PA biosynthetic genes using *ADC, SAMDC, SPDS*, or *SPMS* leads to increased accumulation of free or conjugated PAs, and improves host plant resistance against a wide variety of pathogens including fungi and bacteria. Increase in Put biosynthesis in the host plant without proportionate conversion of Put into Spd and Spm in some cases, increased susceptibility to fungal pathogen. During compatible and incompatible interactions between oat (*Avena sativa* L.) and powdery mildew (*Blumeria graminis* f.sp. *avenae*) pathogen, the susceptible oat cultivar accumulated higher amount of Put vs. the resistant cultivar at early (24 h post-inoculation) infection stage (Montilla-Bascón et al., 2014). Whereas, Spd content was higher in the resistant cultivar in comparison to the susceptible one at the same time point. The results indicate that increase in host Spd production may contribute to resistance against the powdery mildew pathogen. Cotton (*Gossypium hirsutum*) cultivars tolerant to the necrotrophic fungal pathogen, *Verticillium dahliae*, showed greater increase in Spd and Spm content post-fungal inoculation vs. susceptible cultivars, indicating the contribution of higher PAs in resistance against the fungus (Mo et al., 2015). In the present study, TZAR102 and MI82 maize lines previously shown to have resistance to *A. flavus* infection and aflatoxin production (Brown et al., 2016), accumulated higher amounts of Spd and Spm as compared to the susceptible line at the earliest time tested after inoculation (Figure 2B,C). High basal accumulation of PAs in mock-inoculated kernels of the resistant maize lines compared to the susceptible line might suggest the presence of a host defense priming mechanism by polyamines as reported earlier in plants (Hussain et al., 2011). The high Spd to Put ratio in the TZAR102 and MI82 resistant lines compared to the SC212 susceptible line (Figure 2D) indicates a possible role of the host Spd in resistance to aflatoxin production by *A. flavus*. Higher Spd content will contribute to its greater availability for catabolism, conjugation, and other regulatory roles. The mode of action of free Spd and Spm in plant resistance against fungal pathogens has been described through their interactions with PAOs, antioxidant systems, and defense-signaling pathways as reported in several studies (Koç, 2015; Takahashi, 2016; Pal and Janda, 2017; Seifi and Shelp, 2019). Higher Spd/Put ratio in the host plant could also potentially affect uptake of PAs by the fungus especially, Put uptake by *A. flavus* from the maize kernels during pathogenesis. Using radiolabeled PAs in *A. nidulans*, it was shown that the rate of uptake of Put was 2- to 3-fold more rapid than Spd and higher concentrations of Put inhibited Spd uptake (Spathas et al., 1982). Significant increase in the expression of putative Put uptake transporters in *A. flavus* during kernel infection of a susceptible maize variety indicates that uptake of diamine by the fungus may contribute to increased pathogenicity (Majumdar et al., 2018). Besides the role of free PAs in plant pathogen resistance, specific conjugates of Put and Spd have been demonstrated to possess antimicrobial properties in addition to their aid in reinforcing plant cell walls during pathogen infection (Takahashi, 2016; Pal and Janda, 2017). The most resistant line TZAR102 showed up to 250% higher accumulation of the PA conjugate FP, at early and later stages of infection as compared to the SC212 susceptible and MI82 resistant lines used in the present study (Figure 3). Our results on FP in *A. flavus* resistance are in line with an earlier report by Mellon and Moreau (2004), where a concentration dependent inhibition of growth of the AF13 strain was demonstrated *in vitro*.

The role of PAs in plant resistance or susceptibility toward fungal pathogens depends on the type of pathogen (biotroph vs. necrotroph), host species, severity of infection, relative abundance and conversion of the different PAs (reviewed in Pal and Janda, 2017). High basal expression of PA biosynthetic and catabolic genes (in mock-inoculated kernels) in the TZAR102 and MI82 resistance lines also suggests a possible defense priming against *A. flavus* infection. Future RNA-seq studies comparing the transcriptome profiles of inoculated and mock-inoculated resistant and susceptible maize lines will add to our understanding of the regulation of other defense-related signaling pathways in relation to the regulation of PA genes. Catabolism of Spd and Spm by PAOs produces H_2_O_2_, which activates mitogen-activated protein kinases (MAPKs), and wound-induced protein kinases (WIPKs) associated with defense-related pathways in plants (reviewed in Hussain et al., 2011; Moschou et al., 2012). Maize has six *PAO* genes (Jasso-Robles et al., 2016). Based on the predicted intracellular localization of the proteins, ZmPAO1 is extracellular, ZmPAO2, 3, and 4 are peroxisomal/endoplasmic reticulum, ZmPAO5 is cytoplasmic, and ZmPAO6 is cytoplasmic/peroxisomal (Jasso-Robles et al., 2016). Among the different *ZmPAO* genes investigated in the current study, the expression of the *ZmPAO5* gene was highest at 3 dpi and *ZmPAO6* at 7 dpi in the resistant vs. the susceptible lines (Figure 6A,B) at the basal level (mock-inoculated kernels). This might indicate a possible role of *ZmPAO5* and *ZmPAO6* in resistance against *A. flavus* during early and late infection stages in the TZAR108 and MI82 lines. Higher basal expression of cytoplasm and peroxisome specific *ZmPAO*s (2, 3, 6) at early and later development stages respectively (Figure 6B) may have implications on back conversion of Spm or tSpm to Spd and production of H_2_O_2_ involved in plant defense responses (Kamada-Nobusada et al., 2008; Alcázar et al., 2010; Liu et al., 2014; Tiburcio et al., 2014; Pál et al., 2015). Polyamine back conversion and its role against a necrotrophic plant pathogen has been reported (Mo et al., 2015). Heterologous over-expression of a cotton *PAO* gene in Arabidopsis significantly increased Spd content (through back-conversion of Spm to Spd) in the transgenic plants and increased resistance against the fungal necrotroph *Verticillium dahliae* (Mo et al., 2015). In another study, the role of PAOs/DAOs in defense response against the necrotrophic fungal pathogen *Botrytis* (*B*.) *cinerea*, was studied in grapevine (Hatmi et al., 2018). Increases in free PAs in the berries followed by osmotic stress and *B. cinerea* infection without increase in PA catabolism (by oxidases) led to increased berry susceptibility. The results presented here along with previous studies indicate that increase in free PAs accompanied by increased PA catabolism improves maize resistance against *A. flavus*.

### Amino Acid Pools Are Significantly Altered in Susceptible vs. Resistant Maize Lines

It can be expected that any major change in cellular PA content would affect the cellular pool of several AAs as the pathways share common substrates such as Glu, Orn, and Arg (Mohapatra et al., 2010; Majumdar et al., 2013, p. 16). Although storage proteins are less favored as carbon sources by *A. flavus* to produce aflatoxins (Mellon et al., 2000, 2002), Liu et al. (2016) showed that specific AAs such as Glu, Asp, and Asn could significantly increase AFB1 production. Given the observation that cellular contents of Glu and Gln were significantly higher in the susceptible line SC212 than the other lines, it is likely that these two AAs may be responsible for higher aflatoxin accumulation (Figure 4A,B).

Several other AAs including Ala, Pro, and GABA have also been positively correlated with aflatoxin production both *in planta* and *in vitro* studies (Gupta et al., 1977; Payne and Hagler, 1983; Falade et al., 2018). Among these, Pro and GABA are widely associated with abiotic and biotic stress responses and tolerance in plants (reviewed in Hayat et al., 2012; Shelp et al., 2017); both these AAs are closely associated with the PA biosynthetic pathway. The resistant line TZAR102 showed relatively higher Pro content than SC212 at early and late infection stages (Figure 4D). The TZAR102 line is of African origin and associated with drought tolerance (Brown et al., 2016) which might account for its relatively higher Pro content than the SC212 susceptible line. Cellular content of non-protein AA, i.e., GABA, significantly increases in response to diverse stresses and contributes to stress tolerance in plants (reviewed in Shelp et al., 2017).

Proline and GABA are commonly co-induced in many plants in response to various forms of abiotic stress, and both use Glu as the primary substrate. However, the relative proportion of the two is quite different in most cases (Templer et al., 2017; Lawas et al., 2018; Kumar et al., 2019). Stress-induced GABA production in plants has been reported to stimulate fungal pathogenicity (reviewed in Oliver and Solomon, 2004). Consistent with the previous reports, the SC212 line produced >40% higher amount of GABA than the resistant lines during *A. flavus* infection both at early and late infection stages (Figure 4F). In fact, increase in cellular content of GABA was proportionate to the increase in Put content (Figure 2A) in response to *Af*-inoculation. A concurrent increase in cellular content of GABA and increase in aflatoxin production (Figure 4F, 8) in the current study is in line with an earlier report where GABA accumulation was high during infection of maize seeds at different developmental stages (Falade et al., 2018). This may indicate that the production of Pro may be more important for lowering aflatoxin production than GABA. This is consistent with the observation that Orn (substrate for Pro and Put) content in the *Af*-resistant lines (Figure 4C) was lower than that in the susceptible line. Lower Orn content in both the resistant lines (Figure 4C) indicates increased utilization of Orn (either directly through ODC pathway or via ADC pathway) to produce PAs. The observation that *Af*-inoculated TZAR102 and M182 resistant lines had significantly higher content of Orn as compared to their mock-inoculated counterparts on 7 dpi, provides further support to this argument.

Among other AAs, a decrease in Ile and Leu catabolism was associated with a reduction in aflatoxin production in *A. flavus in vitro* studies (Chang et al., 2015). In the present study, with the exception of His, Ser, and Cys at 7 dpi, the content of most AAs were lower in the TZAR102 and MI82 resistant lines compared to SC212 line at 3 and 7 dpi (Figure 4 and Supplementary Figure S1). This could be because large quantities of Glu are being driven toward the synthesis of PAs.

Higher cellular content of specific AAs in the plant (such as Glu, and GABA) coupled with observed increase in aflatoxin levels during infection, noted in the present and previous studies, indicate a possible role for these AAs in susceptibility of the plant to aflatoxin contamination. However, the exact role of specific host plant AAs in aflatoxin production by the fungus can only be delineated through down-regulation of key genes involved in AAs biosynthesis in the plants. Even if the beneficial effects of specific AAs (e.g., GABA) mentioned above are known to improve stress tolerance in plants, their absolute amounts and regulatory roles may vary depending upon the type of environmental stress, abiotic or biotic. There might also be a threshold beyond which excess AAs produced by plants during infection might favor the pathogen through uptake of AAs by the pathogen from the host (Struck, 2015). From a nutritional perspective, several of the AAs discussed above are highly desired in maize seeds. Therefore, future strategies of selection or metabolic engineering of these AAs must be aimed toward balancing the two aspects (need for higher quantities vs. contribution to pathogen susceptibility) of their metabolism in developing *Af*-resistant maize varieties, without compromising the nutritional value of the product in important food and feed crop.

### Polyamines Modulate Aflatoxin Production During Host–Pathogen Interaction

**FIGURE 9:**
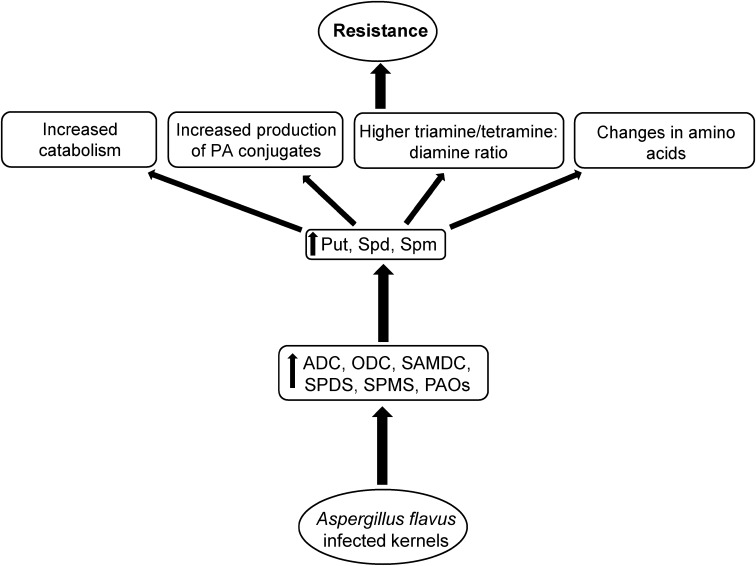
Proposed mechanism of polyamine (PA) mediated *A. flavus* resistance in maize. Kernel infection of *A. flavus* resistant maize genotypes results in up-regulation of PA biosynthetic and catabolic genes, and increases cellular PAs. This in turn is followed by increased PA catabolism, production of specific PA conjugates, and specific AAs that might be associated with increased *A. flavus* resistance and reduced aflatoxin production in the resistant maize genotypes.

A relationship between PA metabolism and aflatoxin production has previously been reported (Khurana et al., 1996; Jin et al., 2002; Khatri and Rajam, 2007; Majumdar et al., 2018). An association with Put accumulation (along with GABA accumulation; which is a direct product of Put catabolism) and aflatoxin production is evident in the susceptible line SC212 compared to the resistant lines, TZAR102 and MI82. High Put accumulation at a later stage of infection (7 dpi) in SC212 (Figure 2) was highly correlated with increased aflatoxin accumulation (Figure 8). Polyamines are common to both plants and their pathogens, and an increase in PA biosynthesis by the host plant during pathogen infection can be advantageous to the pathogen as they can take up PAs (an excellent source of N as well as promoters of growth) that are produced by the host. Using radiolabeled substrates it was shown that Put uptake was more efficient than Spd and Spm uptake in *A. nidulans* (Spathas et al., 1982). As Put levels increased several-fold in the susceptible line at later infection stages (Figure 2A) in the present study, it can be argued that the fungus predominantly takes up Put from the host plant. Upregulation of Put transporters in *A. flavus* during maize kernel infection has been recently reported (Majumdar et al., 2018). Uptake of plant PAs by the invading pathogen was supported by the observation that application of a fungal PA uptake/transport inhibitor reduced *Fusarium graminearum* infection and decreased DON production in wheat by >100-fold (Crespo-Sempere et al., 2015). The results presented here along with the observations of Valdes-Santiago et al. (2012) suggest that Put is an inducer of aflatoxin production in *A. flavus*. Gardiner et al. (2009, 2010) found a similar role of Put in mycotoxin production in wheat-*Fusarium graminearum* pathogenic interaction. The levels of PA pathway intermediates were strongly correlated with the production of deoxynivalenol (DON). Putrescine increased DON production *in vitro* by up-regulating the expression of the biosynthetic genes involved in DON production (Gardiner et al., 2009). The reduction in aflatoxin content in the resistant lines is possibly due to the reduction in fungal load (Figure 7, 8). Similar correlation between fungal load and aflatoxin production in response to infection of maize kernels infection has also been reported in several recent studies (Gilbert et al., 2018; Lebar et al., 2018; Majumdar et al., 2018). The reduction in fungal load in the resistant lines could be a combinatorial result of an increase in free PAs, PA-conjugates, reduction in Orn (Figure 9) along with other genetic factors such as kernel pericarp wax (Maupin et al., 2003). The data presented here suggest that the observed resistance in the TZAR102 and MI82 maize lines to *A. flavus* in maize lines can in part be ascribed to the higher amounts of Spd and Spm rather than increased Put accumulation during *A. flavus* infection.

## Conclusion

Biosynthesis of PAs is critical to both plants and their pathogens, for promoting stress tolerance and pathogenicity, respectively. Equally important is the role of PA catabolism in host plant resistance against pathogens. The current work shows the role of the diamine Put and the higher PAs, Spd and Spm, in susceptibility and resistance of maize to *A. flavus* infection and aflatoxin accumulation, respectively. In general, high basal expression of genes involved in PA biosynthesis and catabolism in absence of the pathogen and their induction upon fungal infection was observed in the resistant lines in comparison to the susceptible line. The data presented here indicate that higher Spd and/or Spm content in maize genotypes may have implication for higher resistance to *A. flavus* and aflatoxin contamination. It should be noted, as mentioned above, that PA metabolism might not be the only factor contributing to resistance against *A. flavus* infection and aflatoxin accumulation in the TZAR102 and MI82 maize lines. Involvement of other metabolites, differences in the molecular genetics of defense responses and physical attributes of kernels, such as waxy seed coat in MI82 (Maupin et al., 2003), are additional characteristics that are likely to contribute to the overall *Af*-resistance in these lines. Future studies focused on analysis of global gene expression along with targeted metabolomics approaches using these resistant and susceptible maize lines will allow for a greater understanding of the mechanisms of PAs in host plant defense responses and *A. flavus* pathogenicity.

## Data Availability

All datasets generated for this study are included in the manuscript and/or the Supplementary Files.

## Author Contributions

RMa, KR, and JC conceived and designed the experiments. RMa performed the experiments. RMa, RMi, SM, ML, SL, and CC-W analyzed the data. RMa and ML wrote the manuscript. RMi, KR, SL, SM, and JC edited the draft manuscript. All authors reviewed and approved the final manuscript.

## Conflict of Interest Statement

The authors declare that the research was conducted in the absence of any commercial or financial relationships that could be construed as a potential conflict of interest.

## Supplementary Material

FIGURE S1 Amino acids are differentially regulated in the *A. flavus* susceptible vs. resistant lines. Cellular contents of **(A)** histidine, **(B)** serine, **(C)** cysteine, **(D)** phenylalanine, **(E)** tryptophan, **(F)** leucine, **(G)** alanine, **(H)** valine, **(I)** isoleucine, **(J)** lysine, **(K)** glycine, **(L)** methionine, **(M)** threonine, **(N)** aspartic acid at 8 h, 3 d, and 7 d in the mock-inoculated (mock-inoc) and *A. flavus* inoculated (*Af*-inoc) kernels of susceptible (SC212) and resistant (TZAR102, MI82) maize genotypes. Data are Mean ± SE of 4 replicates, each replicate consists of 6 seeds (^∗^*P* ≤ 0.05, between the susceptible line SC212 and other lines; ^#^*P* ≤ 0.05, between mock and +*Af* treatments within each line at different times after inoculation).

TABLE S1 Oligonucleotide primers used for the qRT-PCR work.

## Abbreviations

ADC: arginine decarboxylase
ADI: agmatine deiminase
AL: argininosuccinate lyase
Ala: alanine
AA: amino acid
Arg: arginine
AS: argininosuccinate synthase
Asp: aspartate
CPA: *N*-carbamoylputrescine amidohydrolase
Cys: cysteine
DAO: diamine oxidase
dcSAM: decarboxylated *S*-adenosylmethioninie
GABA: γ-aminobutyric acid
Glu: glutamate
Gly: glycine
His: histidine
Ile: isoleucine
Leu: leucine
Lys: lysine
CS: *N′,N″*-di-coumaroyl Spd
FP: *N′,N″*-di-feruloyl-Put
Orn: ornithine
ODC: ornithine decarboxylase
PA: polyamine
OTC: ornithine transcarbamoylase
P5C: Δ^1^-pyrroline-5-carboxylate
PAO: polyamine oxidase
PCA: perchloric acid
Pro: proline
Put: putrescine
SAMDC: *s*-adenosylmethionine decarboxylase
Ser: serine
SM: secondary metabolite
Spd: spermidine
Spm: spermine
SPDS: spermidine synthase
SPMS: spermine synthase
SSAT: Spd and Spm *N*^1^-acetyl transferase
Thr: threonine
Trp: tryptophan
tSPMS: thermospermine synthase
Val: valine
WT: wild-type
