# Supplementary material for: Contribution of Maize Polyamine and Amino Acid Metabolism Toward Resistance Against Aspergillus flavus Infection and Aflatoxin Production
Source: Front Plant Sci. 2019 May 24;10:692. doi: 10.3389/fpls.2019.00692 (PMC6543017; doi:10.3389/fpls.2019.00692)
Supplement: FIGURE S1 — Amino acids are differentially regulated in the A. flavus susceptible vs. resistant lines. Cellular contents of (A) histidine, (B) serine, (C) cysteine, (D) phenylalanine, (E) tryptophan, (F) leucine, (G) alanine, (H) valine, (I) isoleucine, (J) lysine, (K) glycine, (L) methionine, (M) threonine, (N) aspartic acid at 8 h, 3 d, and 7 d in the mock-inoculated (mock-inoc) and A. flavus inoculated (Af-inoc) kernels of susceptible (SC212) and resistant (TZAR102, MI82) maize genotypes. Data are Mean ± SE of 4 replicates, each replicate consists of 6 seeds (∗P ≤ 0.05, between the susceptible line SC212 and other lines; #P ≤ 0.05, between mock and +Af treatments within each line at different times after inoculation). [file Data_Sheet_1.PDF]

Table S1. Oligonucleotide primers used for the qRT-PCR work.

Primer name	Sequence (5'-3')
AFLA_068620 (*β-tubulin*)-qF	AGAGCAAGAACCAGACCTAC
AFLA_068620 (*β-tubulin*)-qR	GACGGAACATAGCAGTGAAC
*Zm*_NM_001148682 (*Odc1*)-qF	TTCCGCATTGACAACGTCCT
*Zm*_NM_001148682 (*Odc1*)-qR	TTGCACTTGACGGCGTAGTA
*Zm*_XM_008672579 (*Odc2*)-qF	CATGCATGCTGCAGAGCAAA
*Zm*_XM_008672579 (*Odc2*)-qR	AACCCAAGTCCGAAGGCAAT
*Zm*_XM_008654778 (*Odc3*)-qF	CCCCGGCGATAAAACCTACA
*Zm*_XM_008654778 (*Odc3*)-qR	TCTCTGGCAGAAGGTACCCG
*Zm*_NM_001323076 (*Adc1*)-qF	GCTACGGCTCAAGGTACCAG
*Zm*_NM_001323076 (*Adc1*)-qR	CCGAACTCCACAATGTCCTC
*Zm*_NM_001138726 (*Adc2*)-qF	GTTCCCCATGATCCTTCGCT
*Zm*_NM_001138726 (*Adc2*)-qR	CCAGGTTGCAGTAGATGCCA
*Zm*_XM_023300699 (*Adc3*)-qF	CCAGATGTTGCTCTCCTTCAACT
*Zm*_XM_023300699 (*Adc3*)-qR	AAAAGTACCAACGGCGGCGA
*Zm*_XM_008671538 (*Adc4*)-qF	TGTCCTCCTGCTCAGAACCG
*Zm*_XM_008671538 (*Adc4*)-qR	AACTGGCGGGTTCCAGAATA
*Zm*_NM_001156222 (*Samdc1*)-qF	GCCTTCCCCACACAAGAACT
*Zm*_NM_001156222 (*Samdc1*)-qR	ACATAAGCATTGCCACCGGA
*Zm*_NM_001112243 (*Samdc2*)-qF	TTGCCAAAGAATTCCCTCCCC
*Zm*_NM_001112243 (*Samdc2*)-qR	TAGTACTCGGCCAGTTCCTCG
*Zm*_NM_001155794 (*Samdc3*)-qF	TGGGCTACCGGCAATTTGTT
*Zm*_NM_001155794 (*Samdc3*)-qR	TGCAGCTCACTATGGCAGAC
*Zm*_NM_001155467 (*Samdc4*)-qF	AGGTGGGATCCTCATCTACCA
*Zm*_NM_001155467 (*Samdc4*)-qR	ATTCTCGCCATCAAAGCAGC
*Zm*_NM_001155838 (*Spds1*)-qF	GCCAAGAGGGAGCTAGAAGC
*Zm*_NM_001155838 (*Spds1*)-qR	GCCGCAGTTAGCTTTTGTGG
*Zm*_NM_001147319 (*Spds2*)-qF	CCAACTCCAGAAGTCCTCCC
*Zm*_NM_001147319 (*Spds2*)-qR	CGAGGGAGGAACGGAGAAAG
*Zm*_NM_001155814 (*Spds3*)-qF	TGCCTGGTGCAGCTATGAAA
*Zm*_NM_001155814 (*Spds3*)-qR	GTGGAAGCGGAAACACAACC
*Zm*_NM_001112372 (*Spms1*)-qF	AAAGGGAGGTGCGGATGATG
*Zm*_NM_001112372 (*Spms1*)-qR	TATCATGGCAGTTGGCCTCG
*Zm*_XM_008664907 (*Spms2*)-qF	GTCGTTGAGTTGGACCCCTT
*Zm*_XM_008664907 (*Spms2*)-qR	TGCCATCTCCCAAATGGACC
*Zm*_NM_001111636 (*Pao1*)-qF	GAAAGTATGACTAGGAAGCTACAG
*Zm*_NM_001111636 (*Pao1*)-qR	GAATAATGAAAAGGCATATGCCGC
*Zm*_NM_001323613 (*Pao2*)-qF	CGGCTCTAAAAGCAGACGAG
*Zm*_NM_001323613 (*Pao2*)-qR	CAAGTAACGCCAGGCACATG
*Zm*_NM_001329439 (*Pao3*)-qF	CTAAGCAGACGAGAGGTTGTC
*Zm*_NM_001329439 (*Pao3*)-qR	GCACATTCCTCATGCCGAAG
*Zm*_NM_001176693 (*Pao4*)-qF	GATATCCAGGTTGTAAGAGAGAATC
*Zm*_NM_001176693 (*Pao4*)-qR	CTGACGAAAAACAGCATCCACC
*Zm*_NM_001175862 (*Pao5*)-qF	CTTAGGTTGCAGACTTGCAG
*Zm*_NM_001175862 (*Pao5*)-qR	CCAAATCCATATACCTTAGCAGT
*Zm*_NM_001137032 (*Pao6*)-qF	AGGAATGCAGGAAGCGGCTC
*Zm*_NM_001137032 (*Pao6*)-qR	GTGATCCAATCTCACTCAGGT
*Zm*_NM_001158589 (*Rib*)-qF	GGCTTGGCTTAAAGGAAGGT
*Zm*_NM_001158589 (*Rib*)-qR	TCAGTCCAACTTCCAGAATGG

Results:

Cellular content of amino acids (Supplementary Fig. S1) that are not direct products of the Glu-Orn-Arg-Pro-Put pathway are described below:

I. Histidine and Ser were lower in the mock- and *Af*-inoculated kernels of TZAR and MI82 lines (vs. SC212) at 3dpi (Fig. S1; A, B). Cellular content of Ser was also lower in the mock-inoculated kernels of TZAR102 and MI82 lines at 8 hpi (Fig. S1, B). No major differences in His and Ser content were observed among samples at 7 dpi.

II. Cysteine was lower in both mock-inoculated and *Af*-inoculated kernels of TZAR102 and MI82 lines at 3 dpi in comparison with SC212 (Fig. S1, C). TZAR102 had the highest amount of Cys in the mock-inoculated samples at 7dpi.

III. Due to poor separation and resolution in the HPLC analysis of Phe and Trp in 8 hpi and 3 dpi samples, only 7 dpi data for these two amino acids are presented here. Phenylalanine and Trp were both lower in the TZAR102, and MI82 lines at 7 dpi in the *Af*-inoculated kernels (Fig. S1; D, E). Leucine was ≥ 80% lower in both mock- and *Af*-inoculated kernels of TZAR102, and MI82 lines at 3 d and in the *Af*-inoculated samples of the same lines at 7 d (Fig. S1, F).

IV. The branched chain amino acids, Ala, Val and Ile, were lower in the mock-inoculated and *Af*-inoculated kernels of TZAR102, and MI82 lines at both 3 and 7 dpi (Fig. S1; G-I). Ile was relatively lower in the resistant lines (vs. susceptible line) at 3 dpi in both inoculants.

V. Cellular Lys and Gly content were lower in TZAR102, and MI82 lines (vs. SC212) at most time points included in this study both in the mock-inoculated and *Af*-inoculated kernels (Fig. S1; J, K).

VI. Methionine was lower in the mock-inoculated kernels of TZAR102 and MI82 lines at 8 hpi, and at 3 dpi decreased by ≥ 80% in both mock-inoculated and *Af*-inoculated samples of the resistant lines as compared to the SC212 susceptible line (Fig. S1, L). At 7 d, no major differences in Met content was observed among the samples. Threonine content was lower in the mock-inoculated samples of TZAR102 and MI82 lines at 8 h and both mock and *Af*-inoculated samples of the same lines at 3 d (Fig. S1, M). At 7 d all lines showed lower Thr content in the *Af*-inoculants in comparison with corresponding mock-inoculants.

VII. No major change in Asp was observed among the samples except at 7 d where Asp was lower in the *Af*-inoculated samples of all lines as compared to corresponding mock-inoculated samples (Fig. S1, N).

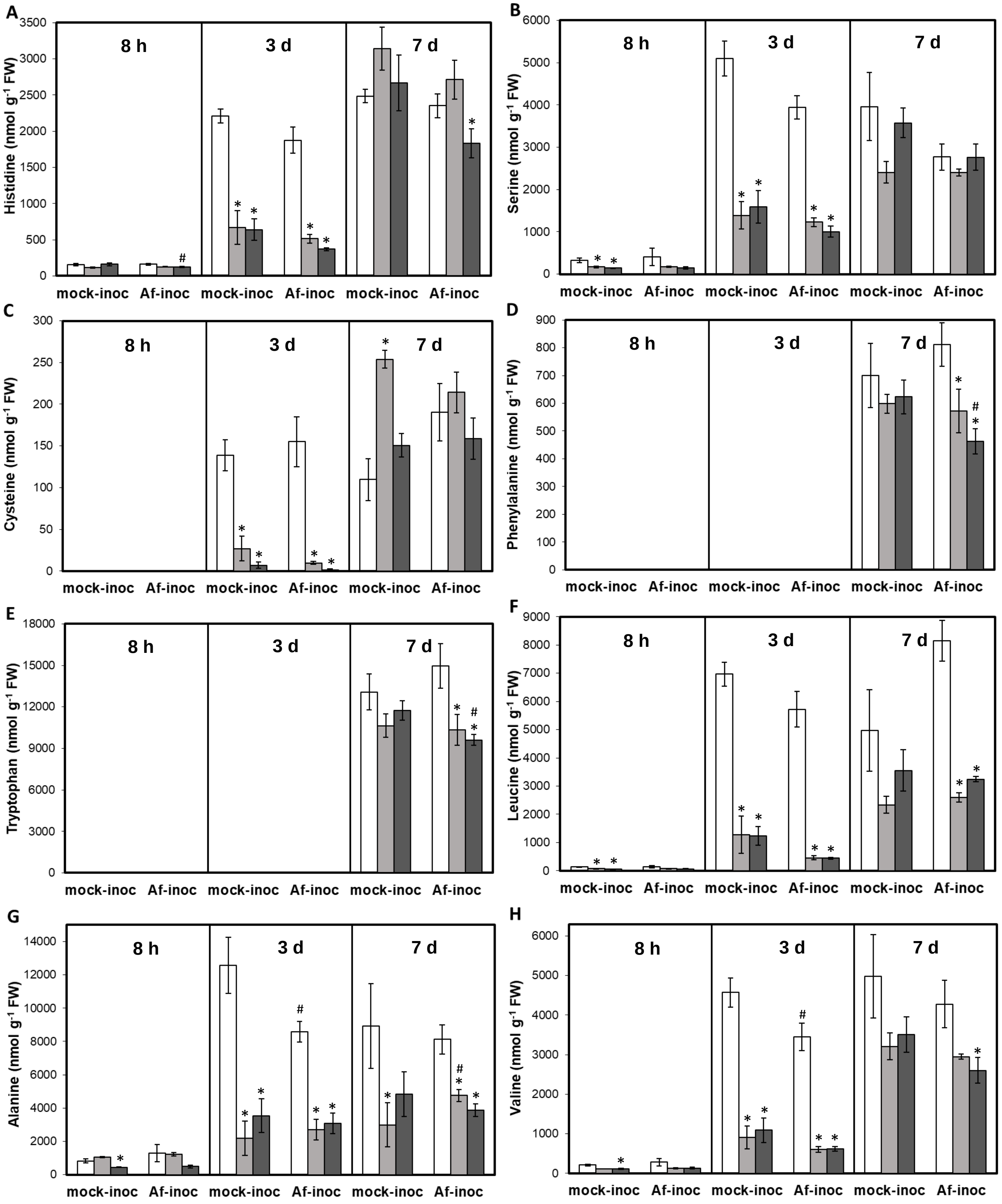
A
Histidine (nmol g-1 FW)
B
Serine (nmol g-1 FW)
C
Cysteine (nmol g-1 FW)
D
Phenylalanine (nmol g-1 FW)
E
Tryptophan (nmol g-1 FW)
F
Leucine (nmol g-1 FW)
G
Alanine (nmol g-1 FW)
H
Valine (nmol g-1 FW)
8 h
3 d
7 d
mock-inoc
Af-inoc

FIGURE S1.

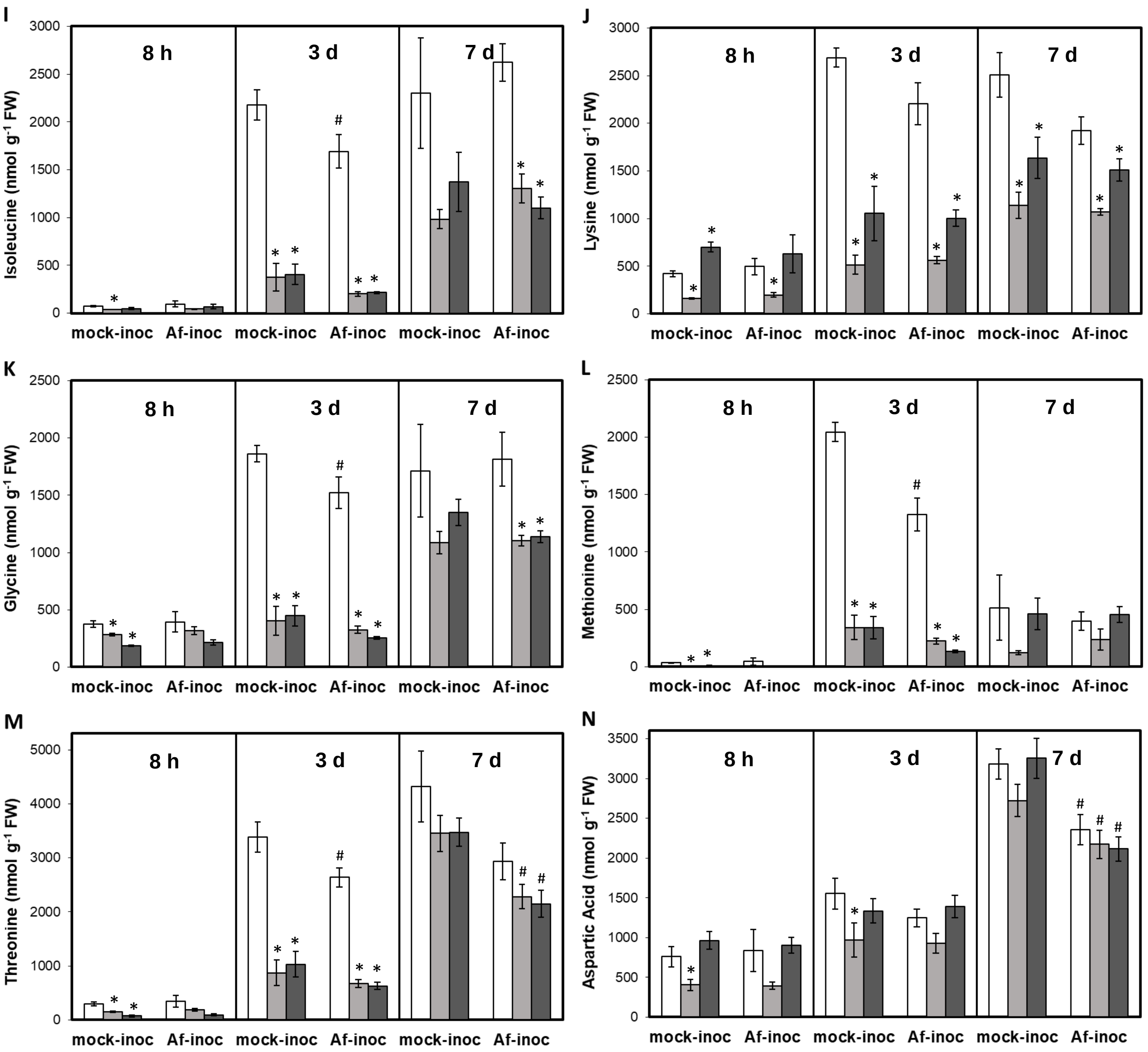

FIGURE S1 (continued)
